# Human papillomavirus genotyping by Linear Array and Next-Generation Sequencing in cervical samples from Western Mexico

**DOI:** 10.1186/s12985-015-0391-4

**Published:** 2015-10-06

**Authors:** María Guadalupe Flores-Miramontes, Luis Alberto Torres-Reyes, Liliana Alvarado-Ruíz, Salvador Angel Romero-Martínez, Verenice Ramírez-Rodríguez, Luz María Adriana Balderas-Peña, Verónica Vallejo-Ruíz, Patricia Piña-Sánchez, Elva Irene Cortés-Gutiérrez, Luis Felipe Jave-Suárez, Adriana Aguilar-Lemarroy

**Affiliations:** División de Inmunología, Centro de Investigación Biomédica de Occidente (CIBO)-Instituto Mexicano del Seguro Social (IMSS), Sierra Mojada No. 800, Col. Independencia, 44340 Guadalajara, Jalisco Mexico; Programa de Doctorado en Ciencias Biomédica, Centro Universitario de Ciencias de la Salud (CUCS), Universidad de Guadalajara, Jalisco, Mexico; Diagnostics Division, Roche México, Mexico City, Mexico; Unidad de Investigación Médica en Epidemiología Clínica, UMAE Hospital de Especialidades, Centro Médico Nacional de Occidente (CMNO)-IMSS, Guadalajara, Jalisco Mexico; Centro de Investigación Biomédica de Oriente (CIBIOR)—IMSS, Metepec, Puebla Mexico; Laboratorio de Oncología Molecular, Unidad de Investigación Médica en Enfermedades Oncológicas (UIMEO)—IMSS, Mexico City, Mexico; Centro de Investigación Biomédica del Noreste (CIBIN)—IMSS, Monterrey, Nuevo León Mexico

**Keywords:** HPV genotyping, Linear Array, 454 Next-Generation Sequencing, PGMY09/11, Human papillomavirus, Mexican women

## Abstract

**Background:**

The Linear Array® (LA) genotyping test is one of the most used methodologies for Human papillomavirus (HPV) genotyping, in that it is able to detect 37 HPV genotypes and co-infections in the same sample. However, the assay is limited to a restricted number of HPV, and sequence variations in the detection region of the HPV probes could give false negatives results. Recently, 454 Next-Generation sequencing (NGS) technology has been efficiently used also for HPV genotyping; this methodology is based on massive sequencing of HPV fragments and is expected to be highly specific and sensitive. In this work, we studied HPV prevalence in cervixes of women in Western Mexico by LA and confirmed the genotypes found by NGS.

**Methods:**

Two hundred thirty three cervical samples from women Without cervical lesions (WCL, *n* = 48), with Cervical intraepithelial neoplasia grade 1 (CIN I, *n* = 98), or with Cervical cancer (CC, *n* = 87) were recruited, DNA was extracted, and HPV positivity was determined by PCR amplification using PGMY09/11 primers. All HPV- positive samples were genotyped individually by LA. Additionally, pools of amplicons from the PGMY-PCR products were sequenced using 454 NGS technology. Results obtained by NGS were compared with those of LA for each group of samples.

**Results:**

We identified 35 HPV genotypes, among which 30 were identified by both technologies; in addition, the HPV genotypes 32, 44, 74, 102 and 114 were detected by NGS. These latter genotypes, to our knowledge, have not been previously reported in Mexican population. Furthermore, we found that LA did not detect, in some diagnosis groups, certain HPV genotypes included in the test, such as 6, 11, 16, 26, 35, 51, 58, 68, 73, and 89, which indicates possible variations at the species level.

**Conclusions:**

There are HPV genotypes in Mexican population that cannot be detected by LA, which is, at present, the most complete commercial genotyping test. More studies are necessary to determine the impact of HPV-44, 74, 102 and 114 on the risk of developing CC. A greater number of samples must be analyzed by NGS for the most accurate determination of Mexican HPV variants.

**Electronic supplementary material:**

The online version of this article (doi:10.1186/s12985-015-0391-4) contains supplementary material, which is available to authorized users.

## Background

Cervical cancer (CC) is the most frequent female cancer worldwide, occupying fourth place among cancers in women. In Mexico, the incidence of this pathology is high (13,960 new cases each year), reaching second place for cancers in women and causing 4769 deaths each year according to Globocan 2012 [1]. Infection with HPV is the principal etiological factor involved in the development of CC [2]. To date, >202 HPV genotypes have been characterized [3] and >40 have been reported to infect the female genital tract [4, 5]. Globally, the HPV genotypes mainly associated with CC include HPV-16, 18, and 45 [6], but these data vary depending on the geographic area. For example, while HPV-35 and 45 are more prevalent in Africa, the genotypes 52 and 58 are more frequently observed in Asia, whereas 33 and 31 are more prevalent in Europe and the USA [7–9]. Regarding Cervical intraepithelial neoplasia grade 1 (CIN I), HPV-42 and 84 were reported as more prevalent in Sweden [10], while in Chile, HPV-61 and 89 [11] are most prevalent, and in Asia, HPV-52 [12] and in the USA (in rural American Indian women), HPV-61 [13]. In Mexico, a recent study of our research group, using genotyping by Linear Array, show that the HPV genotypes most frequently found in cervical cancers were 16 (62.8 %), 18 (11.6 %), 45 (8.3 %), 52/58 (6.6 %), and 39 (5.8 %). Notably, a high percentage (58–64 %) of coinfections were found in CIN I—CIN III, suggesting that coinfections could contribute to regression or progression of the cervical lesions [14].

All of these data suggest the need to develop detection and prevention strategies consistent with the characteristics of the geographic population.

There are few commercial methods available for HPV genotyping [15], the majority of these are based on PCR and reverse hybridization. However, this approach entertains the drawback of only recognizing HPV genotypes with sufficient dissimilarity for discrimination by probes used in hybridization, allowing the detection of a restricted number of HPV genotypes and not discriminating among nucleotide variants. Next-Generation Sequencing (NGS), or Massive parallel sequencing (MPS), has emerged as a powerful tool for genotyping, possessing the advantage of analyzing many samples at the same time. Recently, this methodology has been used for the genotyping of HPV [16–18]. In Mexico, there are few reports of HPV genotyping by means of LA and none, to our knowledge, employing NGS. In this work, we identified, by LA and NGS, the HPV genotypes infecting the cervixes of woman from Western Mexico.

## Results

### HPV genotyping by Linear Array test

**Fig. 1:**
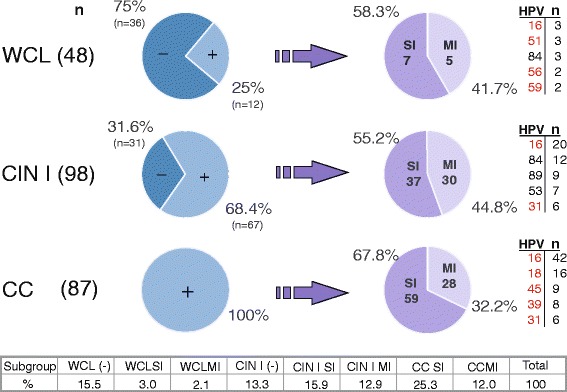
Schematic representation of samples genotyped by Linear Array (LA). This figure depicts the number of patients analyzed in each diagnosis group (*n*), the percentage of Human papillomavirus (HPV) positivity, the percentage of HPV present as Single infection (SI) or Multiple infection (MI), and the top five HPV genotypes present in each group. WCL = Without cervical lesion, CIN I = Cervical intraepithelial neoplasia grade I, CC = Cervical cancer. (−) HPV negative with LA, (+) HPV positive with LA. HPV genotypes carcinogenic to humans are marked in red (International Agency for Research on Cancer [IARC] classification)

From the 48 WCL samples, 12 (25 %) were positive for HPV. Considering only HPV-positive samples, 58.3 % were reported as SI and 41.7 % as MI (Fig. 1). In the CIN I group, 31 samples were found to be HPV-negative (31.6 %) and 67 were HPV-positive (68.4 %). Among the HPV-positive samples, 37 samples were SI (55.2 %) and 30 were MI (44.8 %). In the CC sample group, 100 % (*n* = 87) of the samples were infected with HPV, 67.8 % with SI and 32.2 % with MI.

Calculating the frequency of HPV genotypes detected by LA in the different groups analyzed (Table 1), we observed that the most frequent HPV genotypes found in WCL samples were 16, 51, and 84 (each found in the 25 % of HPV-infected women), followed by 56, 59, and 66 (16.7 % each), and last, HPV genotypes 6, 11, 31, 39, 52, 68, 81, and 89 (8.3 % frequency). Analysis in the CIN I group showed that HPV-16 was the most frequent genotype found in 20 of the 67 HPV-infected patients (29.9 %), followed by genotypes 84 (17.9 %), 89 (13.4 %), 53 (10.4 %), 31 (9 %), and HPVs-39, 56, 58, 61, and 62 (7.5 %). HPV-66 was present in 6 % of the women of this group, 16 additional HPV genotypes were found at frequencies less than 4.5 %, as detailed in Table 1. On the other hand, the CC group exhibited that HPV-16 was also the most frequent genotype found (in 42 of 87 HPV-infected patients, 48.3 %), followed by 18 (18.4 %), 45 (10.3 %), 39 (9.2 %), 31, 52, and 71 (with 6.9 % frequency each), 68 (5.7 %), and 19 additional HPV genotypes with frequencies ranging between 4.6 and 1.1 %. It is noteworthy that HPV-16 was the most frequent genotype found in all analyzed groups. Interestingly, HPV-52, 56, and 68 were found only in patients with MI; and HPV-26, 69, 70, and 72, were detected only in the CC group.

**Table 1:**
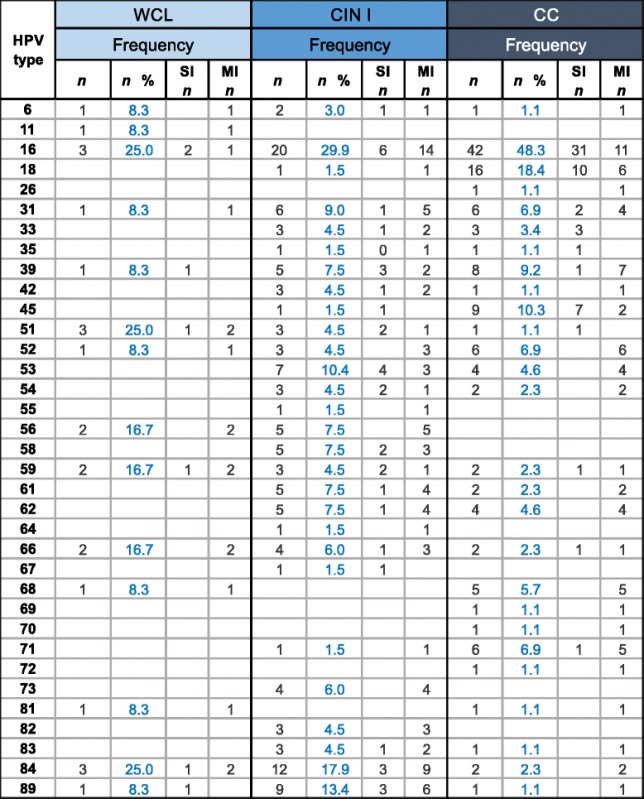
Frequencies of each Human papillomavirus (HPV) genotype identified by Linear Array (LA) according to diagnosis group

The Table shows the number of patients (*n*) and the % of the frequency, in which each HPV genotype was found. WCL (Without cervical lesion), CIN I (Cervical intraepithelial neoplasia grade I), CC (Cervical cancer), SI (Single infection), and MI (Multiple infection)

### HPV genotyping by Next-Generation Sequencing

**Fig. 2:**
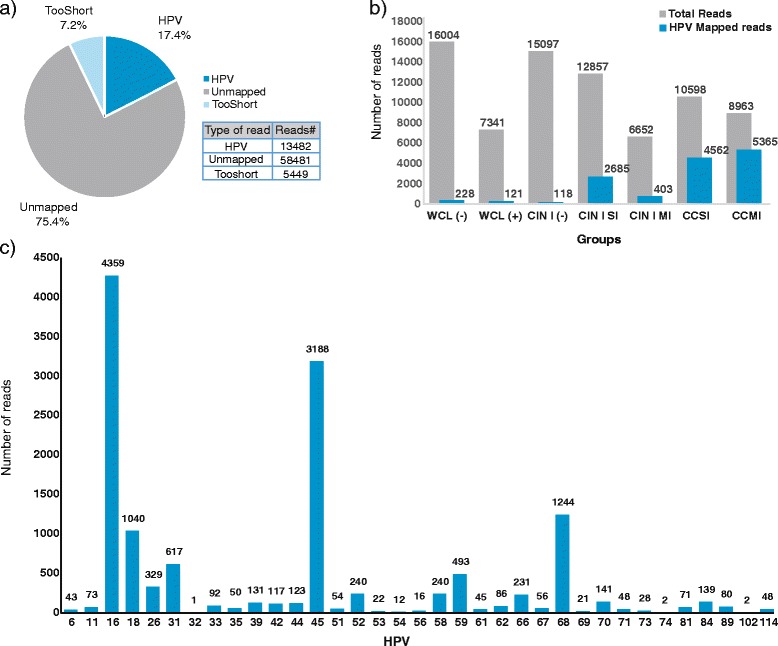
Next-Generation Sequencing (NGS) data. **a** Percentage of total reads obtained in the entire sequencing, grouped as unmapped, Human papillomavirus (HPV)-mapped, or TooShort (eliminated). **b** Diagram of total reads obtained in each pooled study group (*gray*), including HPV mapped reads (*blue*) obtained in each group utilizing the HPV reference sequence from the Papillomavirus Episteme (PaVE) database. **c** Total reads mapped with each HPV genotype independent of the diagnosis group

To corroborate the genotypes detected by LA and to determine additional HPV not included in this test, NGS was performed. All samples positive for HPV by LA, as well as negative for LA but positive with PGMY09/11 primers, were included. Samples were pooled according to diagnosis and SI or MI infection. Samples negative by LA but positive with PGMY09/11 primers were designated as WCL (−) or CIN (−). As depicted in Fig. 2a, after comparison of the sequences obtained with the human L1 papillomavirus database downloaded from PaVE, 75.4 % (58,481) of the reads were reported as unmapped or not recognized as belonging to any reported HPV, 7.2 % (5549 reads) were tooshort (<100 pb), and finally, 17.4 % (13,482 reads) were found to be mapped with HPV sequences. Median length of HPV reads was 450 nucleotides (nt), ranging between 404 and 468, while the modal length of the reads was 460 nt.

Comparing the number of reads obtained by group, it was observed that the greatest number of reads was obtained in the WCL (−) group, followed by the CIN I (−), CIN I SI, CC SI, CC MI, WCL (+), and CIN I MI groups (Fig. 2b). However when we analyzed the proportion of reads belonging to HPV by group, we observed that the CC MI group produced the greatest number of reads, followed by the CC SI, CIN SI, and CIN MI groups. Groups CIN (−), WCL (−), and WCL (+) had low numbers of reads belonging to HPV in relation to the numbers of reads obtained in these groups. Of the total reads that mapped to HPV, 228 sequences belonged to the WCL (−) group, 121 to WCL (+), 118 to CIN I (−), 2685 to CIN I SI, 403 to CIN I MI, 4562 to CC SI, and 5365 to the CC MI group (Fig. 2b). Analyzing the total reads obtained for each HPV genotype, the majority of reads were from HPV-16 (4359 reads), followed by HPV-45 (3188 reads), HPV-68 (1244 reads), HPV-18 (1040 reads), and HPV-31 (617 reads) (Fig. 2c).

As depicted in Table 2, reads for each HPV were also determine according to diagnosis group. In the WCL (−) group (samples WCL negative for HPV by LA), we observed the presence of HPV-26, 32, 44, and 51; of these HPV, it is noteworthy that HPV-26 and 51 are included in the LA test. In the WCL (+) group, nearly all genotypes reported by LA were found, but additionally, HPV-52, 81, and 89 were also found (despite that all of these genotypes are included in the LA test). On the other hand, the presence of HPV-6, 11, 31, 39, 56, and 68, which were determined by LA in this group, was not confirmed by NGS. Regarding the CIN I (−) group, we were able to find, by means of NGS, 85 reads mapping to HPV-16 and 33 reads that mapped to HPV-35, despite that the test included both HPV genotypes. With respect to the CIN I SI group, HPV-44 and 74 were additionally found, genotypes that are not included in LA. Similarly, in the CIN I MI group, additional HPV genotypes that are not included in LA were detected by NGS, such as 44, 102, and 114. In terms of the CC SI group, HPV-11, 58, 68, 73, 81, 89, and 114 were identified by NGS, but not by LA. Among these, the LA test possesses probes for nearly all HPV, but not for HPV-102 nor for HPV-114. Finally, in the CC MI group, HPV-58, 74, 102, and 114 were additionally detected (Table 2). For comparison, all results obtained by LA in each sample analyzed are contained in Additional file 1: Table S1.

**Table 2:**
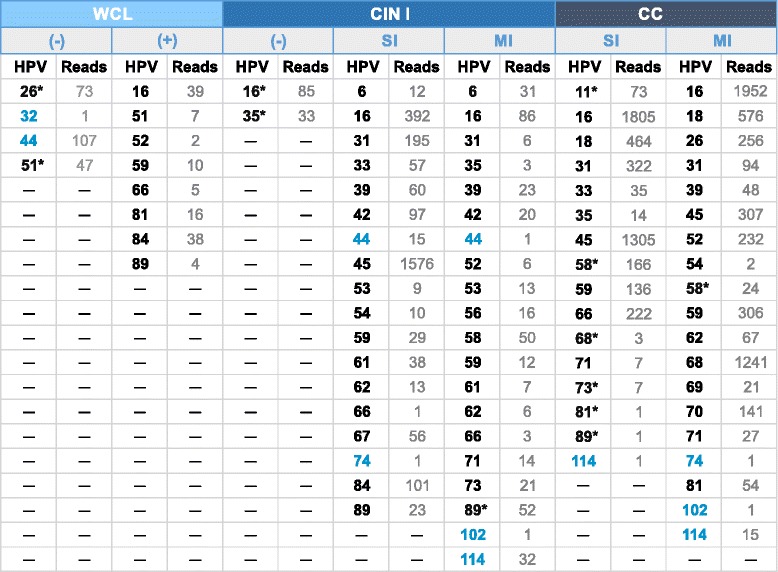
Number of reads obtained for each Human papillomavirus (HPV) genotype according to study group

HPV genotypes in blue are those not recognized by Linear Array (LA). HPV genotypes in black were found by Next-Generation Sequencing (NGS) and LA. HPV genotypes with asterisk are those included in the LA test, but were only recognized by NGS. WCL (Without cervical lesion), CIN I (Cervical intraepithelial neoplasia grade I), CC (Cervical cancer), (−) HPV-negative with LA, (+) HPV-positive with LA, SI (Single infection), and MI (Multiple infection)

For a better overview of all NGS results, a summary of HPV genotypes found according to diagnosis group are depicted in Table 3, in which the HPV genotypes found by LA and NGS are schematized in black; HPV genotypes, in blue, found only by NGS despite their being included in the LA test; and in pink, genotypes found only by NGS that are not included in LA. Those HPV genotypes detected by LA and not by NGS were also included (asterisk). Interestingly, by using NGS, the following genotypes not included in the LA test were identified: HPV-32, 44, 74, 102, and 114. If we analyze the presence of each HPV by diagnosis group, we may observe that HPV-16 was detected in six of the seven analyzed groups, HPV-59 was detected in five groups, HPV-31 and 66 in four groups, and the remainder of HPV genotypes was found in three or two groups. Genotypes identified in only one group comprised HPV-11 in CC SI, 32 in WCL (−), 56 in CIN I MI, 67 in CIN I SI, 69 in CC MI, and 70 in CC MI, as can be observed in Table 3.

**Table 3:**
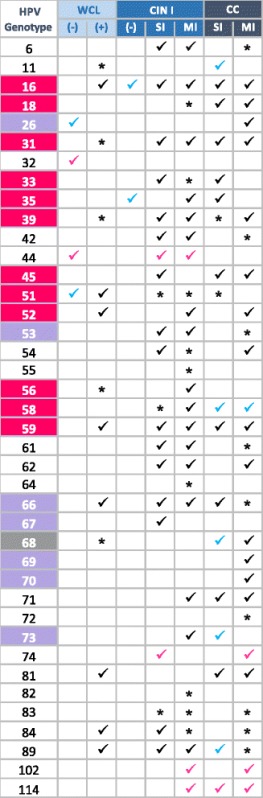
Summary of all Human papillomavirus (HPV) genotypes determined by Next-Generation Sequencing (NGS) in each study group

Pink checkmarks represent HPV genotypes found only by NGS that are not included in Linear Array (LA); blue checkmarks represent HPV genotypes only detected by NGS, despite their being included in LA; black checkmarks are HPV genotypes found both by LA and by NGS. HPV genotypes detected by LA but not by NGS are marked with an asterisk. Boxes in pink represent genotypes reported as “carcinogenic to humans”, in gray as “probably carcinogenic”, and in purple as “possibly carcinogenic” (International Agency for Research on Cancer [IARC] classification)

**Fig. 3:**
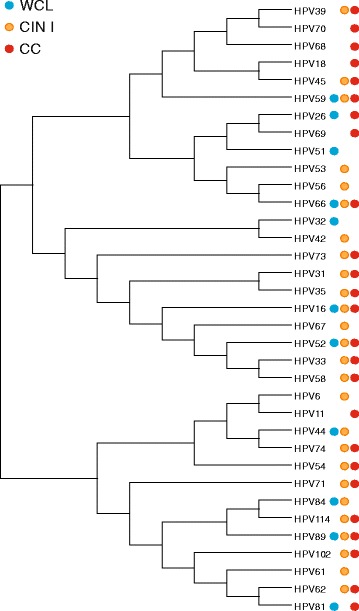
Phylogenetic tree built with the total Human papillomavirus (HPV) consensus sequences identified in Mexican cervical samples analyzed. Analysis was performed by MEGA6 software displayed as Topology. The diagnosis group in which each HPV genotype was detected is also depicted. *Blue circle*: WCL (Without cervical lesion), *Orange circle*: CIN I (Cervical intraepithelial neoplasia grade I), and *Red circle*: CC (Cervical cancer)

Finally, consensus sequences (contigs) of each HPV genotype found exclusively among the Mexican samples analyzed were aligned and a phylogenetic tree was constructed (Fig. 3). As expected, HPV contigs were grouped according to the present classification. Exception included HPV-54, found more closely related with HPV-6, 11, 44, and 74. To supplement these data, next to each genotype is displayed the diagnosis group in which the sequences were found (WCL, CIN I, or CC). To contrast these results, we noted that HPV-11, 18, 68, 69, and 70 were only found in CC, whereas HPV-32 and 51 were only found in the WCL group. On the other hand, HPV-31, 33, 35, 39, 45, 54, 58, 62, 73, 74, 102, and 114 were present in CIN I and CC, whereas HPV-44 and 84 were found in the WCL and CIN I groups.

## Discussion

Cervical cancer remains a public health problem in Mexico, despite the prevention and vaccination campaigns implemented by the government. Vaccination is expected to decrease the incidence of this malignancy in the near future, and this probably would impact differentially, depending on the geographic region, because it has been shown that HPV genotype frequency varies among the different countries [19–22]. Thus, there are local efforts to develop population-specific directed HPV vaccines; however, to develop a vaccine specific for a geographic population, it is essential to first know the HPV genotypes circulating in the population. In this study, we detected the HPV genotypes that are infecting women from Western Mexico, not only by LA, which is the commercial test with the major number of genotypes included, but also by NGS, to identify the widest possible genotypes. Results obtained by LA were very similar to those previously reported and discussed by our IMSS Research Network on HPV [14]; however, the use of 454 NGS allowed us to identify previously not reported HPV genotypes in Mexican population. NGS is a technology that has been efficiently used for HPV genotyping; this methodology is based on Massive Sequencing (MS) of HPV fragments from a great number of genotypes, even when these are in low in copy number [16, 18].

Using 454 NGS, we could detect genotypes not included in LA: 32, 44, 74, 102, and 114 (1, 79, 2, 2, and 30 reads, respectively). We found HPV-32 only in the WCL group; this genotype has been associated with oral focal epithelial hyperplasia [23, 24] and oral papillomatous lesions from patients with Human immunodeficiency virus (HIV) infection [25]. In another study, in a search for uncommon and rare HPV genotypes in cervical carcinomas, HPV-32 was not found [26]. On the other hand, characterization of the properties of HPV-32 E6 and E7 oncoproteins have provided evidences of the benign nature of this genotype [27].

With respect to HPV-44, we detected this genotype in WCL or CIN I samples, but not in CC. This HPV was isolated for the first time from a vulvar condyloma, and was found in five cases of 439 normal squamous epithelium samples and in three cases of 112 CIN I samples, but was not found in any of the 56 CC analyzed [28]. However, this genotype has been found in patients with invasive cervical cancer in Africa with a frequency of 1.8 % of co-infection with other HPV genotypes [29]. Additionally, it has been detected in patients with HIV with a frequency of 12.5 % in South Africa, Ghana, and Nigeria [29], and has been also reported in anogenital carcinoma [30]. In other studies, the prevalence of HPV-44 has been reported as ranging from 0.4–5 % [31–34].

HPV-74 has been found in low-grade vaginal intraepithelial neoplasia in immunosuppressed women and has been related phylogenetically with HPV-6, 11, 44, and 55 [35, 36]. We found this genotype only in some patients of the CIN I SI and CC MI groups. The number of reads obtained for HPV-74 was very low, suggesting a low prevalence. In this regard, low prevalence of HPV-74 has been detected by LiPA assay in genital warts in a German population [34]; additionally, HPV-74 has also been reported as present in male populations from Brazil, Mexico, and the USA (by direct sequencing of products from nested PCR of the L1 region) [37].

HPV-102 has been also found in this study only by NGS; we detected it in CIN I and CC, both in MI (Table 3). Despite that we found only one read in each group, we consider this genotype as present, because the sequences obtained are sufficient (337 and 411 bp) and identity was 99 and 100 %, respectively. Additionally, phylogenetic tree analysis places this genotype in *alpha* species 3 (Fig. 3), as also reported [36]. It is important to mention that we used all HPV reference sequences from the L1 *alpha* species for mapping. It has been reported that “simultaneous mapping” significantly decreases the read numbers obtained [38], but we consider that it is better because more solid data are obtained. In agreement with our findings, this genotype has been detected in cervicovaginal cells from Hispanic females [39].

Other additional genotype detected in our study, only by NGS, was HPV-114; this is a recently characterized papillomavirus isolated from a low-grade cervical lesion, and it has been found in low-grade samples with 1.7 % prevalence [40]. We found this genotype in CIN I MI and CC, both SI and MI; its presence in CC suggest a possible oncogenic risk that needs to be evaluated individually in more samples. HPV-114 has also been detected by NGS in a Swedish cervical sample [17], and has been found in one case of Vulvar intraepithelial neoplasia (VIN) [41].

To complement and summarize our results, it was in our interest to performed a phylogenetic tree exclusively with sequences obtained by NGS from Mexican samples (Fig. 3); reconstruction shows a very similar pattern according to the classification of de Villiers [36] despite using approximately 450 bp of the L1 region amplified by the PGMY09/11 set of primers. Bernard et al. in 1994 demonstrated that a short 291-bp fragment of the *L1* gene is sufficient to generate phylogenetic comparisons [42].

It is accepted that in order to recognize a new Papillomavirus (PV) type, its complete genome must be cloned and its *L1* gene sequence must be different in 10 % from the closest type known; differences between 2 and 10 % represent a subtype, and differences <2 %, a variant [43]. By analysis with the GS Reference Mapper, a variety of putative high-confidence nucleotide differences were determined, principally in HPV genotypes 31, 39, 45, 62, 66, 68, and 69. These nucleotide differences were found in two or more diagnosis groups (taking into account differences detected in at least 50 reads). Therefore, we continue to not discard the existence of HPV variants present in Mexico.

Regarding the classification of carcinogenicity for humans used by the International Agency for Research on Cancer (IARC) [44], there are some HPV genotypes that are classified as “probably” or “possibly” carcinogenic or not classifiable. Among those, we found HPVs-11, 68, 70, and 69 only in CC samples. In contrast, HPVs-32, 44, 51, and 84 were observed in only the WCL and/or in the CIN I group. With regard to HPV genotypes not previously reported in Mexican population, HPV-74, 102, and 114 were detected in the CIN I and CC groups.

One limitation of this study was that groups of samples (according to the diagnosis), but not individually samples, were sequenced. Additionally, the HPV-genotypes detected were limited to the amplicons obtained by using the PGMY09/11 set of primers.

We continue to recruit and analyze additional cervical samples in order to corroborate and report these observations. Genotyping of HPV by NGS will permit the detection of more possible HPV variants, whose sequences could increase or diminish HPV recognition by the immune system or modify the oncogenic potential of HPV proteins [45].

We continue to need more studies with methodologies such as NGS to determine the HPV genotypes and variants circulating in Mexican population and at the different stages during CC progression.

## Conclusions

To our knowledge, this is the first time that data has been reported on HPV genotypes circulating in Mexico by using Massive parallel sequencing (MPS). Our data provide helpful information because we were able to find not previously detected HPV genotypes in Mexican population. The methodological strategy employing the PGMY09/11 primers appears to be a very good system.

## Methods

### Sample collection

Approximately 250 cervical samples were obtained from the Centro Médico Nacional de Occidente (CMNO, IMSS) located in Guadalajara, Jalisco, Western Mexico. The women whose samples were taken attended a medical examination due to a cervical ailment. Diagnosis was first performed only by colposcopy observation (visual inspection using acetic acid and Lugol’s iodine solution); when cervical lesions/cancer were (was) observed, a biopsy was taken for diagnosis confirmation by histopathology. From the 250 samples obtained, 17 samples were excluded (those samples without sufficient quantity and quality of DNA), therefore, 233 samples were included in this study: 48 WCL; 98 CIN I, and 87 CC. Ages of the participating women ranged from 25–65 years. Samples were obtained by inserting a cytobrush into the endocervical canal, rotating it for 3–5 full turns, and placing it into ThinPrep PreservCyt® transport medium solution (Cat. no. 70097–002; Hologic, Inc., Marlborough, MA, USA).

### HPV screening and Linear Array® HPV genotyping test

Total DNA was extracted and purified from each cervical sample using the AmpliLute Liquid Media Extraction Amplicor Kit (Cat. no. 03750540 190; Roche Molecular Systems, Inc., Branchburg, NJ, USA.). A first screening for HPV positivity was performed by conventional single-round PCR utilizing PGMY09/11 primers, which amplified a 450-bp fragment of L1 [46]. HPV-positive samples were genotyped utilizing the Linear Array® HPV Genotyping test (Cat. no. 04391853 190; Roche Molecular Diagnostics, Pleasanton, CA, USA), which is capable of detecting simultaneously up to 37 HPV genotypes in a sample [47]. This assay includes, as control, β-globin amplification in the same PCR reaction. According to the results obtained by LA, samples were subdivided into HPV-negative (−) or HPV-positive (+), and into Single- or Multiple infection (SI or MI, respectively).

### HPV sequencing by 454 NGS

All HPV-positive samples identified by conventional PCR utilizing PGMY09/11 primers were included for MS. Amplicons for the NGS Library were generated by taking approximately 500–1000 ng of DNA from each sample and amplification by conventional PCR employing the following specifically designed primers: *Forward* 5′-[Universal Multiplicom tail A: AAGACTCGGCAGCATCTCCA] – [individually PGMY11 primer]-3′, and *Reverse* 5′-[Universal Multiplicom tail B: GCGATCGTCACTGTTCTCCA] – [individually PGMY09 primer]-3′. The PGMY sequences used were those reported in Table 2 in Gravitt et al. [46]. Universal Multiplicom tails A and B were the same used in Cat. no. MR-0020.024, Multiplicom NV CFTR; Molecular Diagnostics, Niel, Belgium. PCR conditions included 1 cycle at 94 °C for 3 min, followed by 40 cycles at 94 °C for 45 s, 48 °C for 30 s, and 72 °C for 60 s, and a final cycle at 72 °C for 10 min. The PCR products (~450 pb) were electrophoresed in 1.5 % agarose gels, and cut and purified with the Wizard® SV Gel and PCR Clean-Up System (Cat. no. A9282; Promega, Madison, WI, USA).

Purified amplicons generated from each sample were quantified with the Qubit® dsDNA HS fluorometry assay Kit (Cat. no. Q32851; Life Technologies™, Eugene, OR, USA) to carry out 5-ng amplicon pools per sample. Amplicon pools were generated for the NGS Library, taking into account diagnosis, HPV negativity or positivity observed by LA, and the SI- or MI-HPV infection detected in a same sample. The following groups were generated: WCL (−), samples WCL-negative for HPV by LA; WCL (+), samples WCL-positive for HPV by LA; CIN I (−), CIN I samples negative for HPV by LA; CIN I SI, CIN I samples with single HPV infection; CIN I MI, CIN I samples infected with 2 or more HPVs, and CC SI and CC MI.

A second PCR reaction was performed to add the Multiplex identifiers (MID) barcodes, the key sequence (TCAG), and A or B sequencing adapters by using Multiplicom 454 MID Kits (Cat. no. ML-0008.192 and ML-0016.192, Multiplicom NV CFTR; Molecular Diagnostics) according to the manufacturer’s instructions. Amplicons where then purified with Agentcourt® AMPure® XP beads (Cat. no. A63881; Beckman Coulter Genomics, Danvers, MA, USA) and evaluated with the 2100 Bioanalyzer system (Cat. no. G2940CA; Agilent Technologies, Santa Clara, CA, USA).

The emulsion Polymerase chain reaction (emPCR) was performed with the GS Junior Titanium emPCR kit Lib A (Cat. no. 05996520001; Roche Diagnostics), and amplicons were sequenced in a GS Junior Sequencer utilizing the GS Junior Titanium Sequencing Kit (Cat. no. 05996554001) and the GS Junior Titanium PicoTiterPlate Kit (Cat. no. 0599661900), following the manufacturer’s instruction. All kits were supplied by Roche Diagnostics, Basel, Switzerland.

### Data analysis

Quality control for sequencing data was performed, sequences with a Phred quality score of <20 were eliminated, and primer sequences were trimmed. The Roche GS Reference Mapper (ver. 2.9) was used to identify HPV genotypes by comparing fragment sequences with the L1 region of *alpha* and *beta* papillomavirus reference sequences, downloaded from Papillomavirus Episteme (PaVE) [48]. Specific reads for each diagnosis group were identified in accordance with their associated MID barcode.

### Analysis of phylogenic ancestry

Construction of a phylogenetic tree with consensus sequences of each HPV was performed. First, all consensus sequences were aligned utilizing MEGA6 software (employing the ClustalW ver. 1.6 algorithm), followed by the construction of the phylogenetic tree with the maximum likelihood method. Traditional tree-branch style was chosen and the Topology display was selected.

## Ethical approval

This study was approved by the Ethics and Research Committees of the Instituto Mexicano del Seguro Social (IMSS), with registration number R-2014-785-036. All women provided written informed consent before the sample collection.

## Additional file

Additional file 1:
**Table S1.** Human papillomavirus (HPV) genotypes identified by Linear Array (LA). The Table shows the HPV genotypes found in each sample analyzed and grouped according to diagnosis. WCL (Without cervical lesion), CIN I (Cervical intraepithelial neoplasia grade I), CC (Cervical cancer), SI (Single infection), and MI (Multiple infection). (PDF 20 kb)

## Abbreviations

HPV: Human papillomavirus
WCL: Without cervical lesion
CIN I: Cervical intraepithelial neoplasia grade I
CC: Cervical cancer
LA: Linear Array
SI: Single infection
MI: Multiple infection
PaVE: Papillomavirus Episteme
NGS: Next-Generation Sequencing
