# Supplementary material for: Human papillomavirus genotyping by Linear Array and Next-Generation Sequencing in cervical samples from Western Mexico
Source: Virol J. 2015 Oct 6;12:161. doi: 10.1186/s12985-015-0391-4 (PMC4596464; doi:10.1186/s12985-015-0391-4)
Supplement: Additional file 1: — Table S1. Human papillomavirus (HPV) genotypes identified by Linear Array (LA). The Table shows the HPV genotypes found in each sample analyzed and grouped according to diagnosis. WCL (Without cervical lesion), CIN I (Cervical intraepithelial neoplasia grade I), CC (Cervical cancer), SI (Single infection), and MI (Multiple infection). (PDF 20 kb) [file 12985_2015_391_MOESM1_ESM.pdf]

WCL	CIN I		CC		
SI and MI	SI	MI	SI		MI
HPV genotypes detected by LA					
16	45	84, 89, 16	16	35	16, 18, 52
59	31	59, 62	16	16	16, 18
68, 81,84	54	71, 33	18	16	31, 54, 71, 83, 89
51	16	58, 66, 89	16	33	16, 59
6, 11, 16, 59, 84	62	31, 56, 66, 82,84	18	18	18, 42
51, 52, 56, 66	67	6, 53, 61, 84, 89	66	18	39, 68
56, 66	89	31, 39	16	16	45, 72
31, 51, 59	33	16, 53, 62	16	16	39, 68
84	66	62, 89	31	16	16, 31
39	84	16, 84	16	51	16, 62
89	42	16, 42	18	18	16, 18, 62
16	6	35, 84	45	16	16, 54, 62, 70
	16	73, 83	45	16	52, 53, 66, 68
	59	61, 84	33	45	45, 84
	53	16, 18, 31, 33, 58	16	16	52, 53, 68
	84	52, 61	16	16	16, 52
	53	56, 66, 89	45	18	16, 18
	16	16, 52, 82,84	45	16	6, 39
	61	82, 84	71	18	16, 52
	54	16,18, 73	59	16	39, 62, 71
	39	16, 31, 53, 62	16	16	16, 52
	89	16, 42	18	39	69, 71, 81, 84
	16	16, 64	16	16	39, 68
	16	16, 39, 52, 84, 89	16	45	18, 26
	89	56, 73	33	16	31, 53, 61
	84	31, 73	16	16	39, 71
	53	16, 58	45	16	31, 53, 61
	51	54, 55, 61, 83, 89	18	31	39, 71
	58	16, 51, 56	16	16	
	53	6, 16, 56		16	
	39				
	39				
	59				
	83				
	16				
	51				
	58				
